# Scrotal Pyocele Secondary to Perforated Appendicitis in Middle-Aged Man: A Case Report and Review of Literature

**DOI:** 10.7759/cureus.51476

**Published:** 2024-01-01

**Authors:** Ahmed A Al Bazroon, Ali M Alabandi, Murtadha M Alnemer, Shadi Abu Alsaud

**Affiliations:** 1 Department of Urology, Dammam Medical Complex, Dammam, SAU

**Keywords:** patent processus vaginalis, acute appendicitis, scrotal swelling, scrotal abscess, acute scrotum, scrotal pyocele

## Abstract

A scrotal abscess following perforated appendicitis is a rare complication. This is mostly seen in children and usually occurs due to the presence of the patent processus vaginalis, which permits the passage of pus from the intra-abdominal cavity down to the scrotum, resulting in a scrotal pyocele. There are few reported cases of such complications in young adult patients. We report a case of a 50-year-old male with a scrotal pyocele following exploratory laparotomy for perforated appendicitis. Scrotal ultrasound (US) findings were suggestive of a right-sided scrotal abscess, and the CT scan showed prominent fat in the proximal part of the right inguinal canal, which suggested the presence of a partially patent processus vaginalis. Our case is the first reported in the middle-aged group, and our review of the literature is the first directed to the adult age group. This review emphasizes the importance of considering scrotal pyocele in any patient with acute scrotum post-appendectomy, regardless of the patient's age, the affected side, and the presence or absence of identifiable patent processus vaginalis, as it may be microscopically permeable. Treatment will entail urgent drainage of the abscess, together with a course of antibiotics.

## Introduction

Testicle formation starts in the intra-abdominal cavity at seven to nine weeks of gestation. Testicular descending from the abdominal cavity to the scrotum occurs between seven and eight months of gestation and is guided by the gubernaculum. While passing through the inguinal canal, the testicle takes a piece of the peritoneum into the scrotum, which forms the tunica vaginalis (a peritoneal layer that surrounds the testicles), which remains connected to the peritoneal cavity through a duct that extends to the inguinal ring (processus vaginalis).

Spontaneous obliteration of the processus vaginalis occurs with time, and there will be no further communication between the intra-abdominal cavity and the tunica vaginalis. Patent processus vaginalis has been estimated to be present in 80%-95% of newborn males, 60% at one year of age, 40% at two years, and 15%-37% persist into adulthood [1, 2]. Patent processus vaginalis allows the passage of fluid from the intra-abdominal cavity down to the tunica vaginalis, and this may result in congenital hydrocele. This rare condition usually occurs in children and less commonly in young adults.

Among the other rare clinical conditions related to patent processus vaginalis is acute scrotum secondary to intra-abdominal inflammatory collection that results in scrotal pyocele. This condition is very rare; it has been seen mostly with perforated appendicitis in patients with patent processus vaginalis and is mostly seen in the pediatric age group. There were a very limited number of cases reported among young adults. We report a case of a middle-aged male aged 50 years with scrotal pyocele following exploratory laparotomy for perforated appendicitis.

## Case presentation

A 50-year-old male, with no medical or surgical history, presented to the ER with a history of abdominal pain for two days. Pain started in the right lower area of the abdomen, and then became generalized all over the abdomen. Pain was constant, severe, and associated with anorexia, nausea, several episodes of vomiting, constipation, and abdominal distension. On examination, he was tachycardiac, with a distended abdomen, diffuse tenderness, and guarding all over.

**Figure 1:**
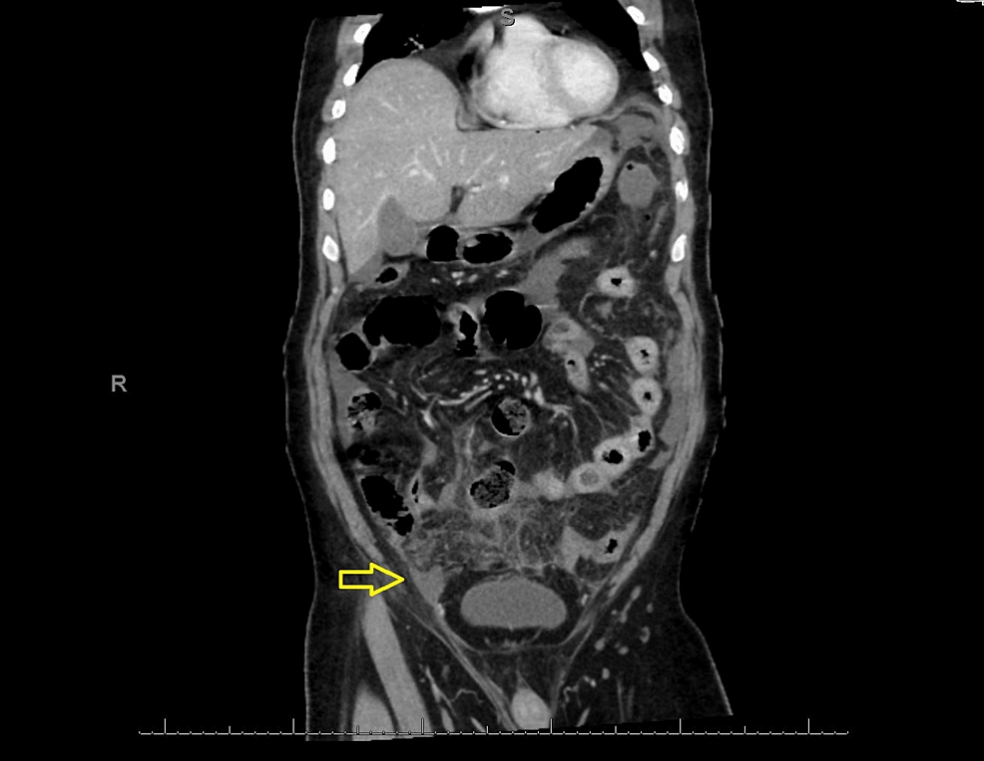
The CT scan shows findings of perforated appendicitis. The area of pus collection is labeled with an arrow on the right, and there is no extension of pus collection into the right inguinal canal.

**Figure 2:**
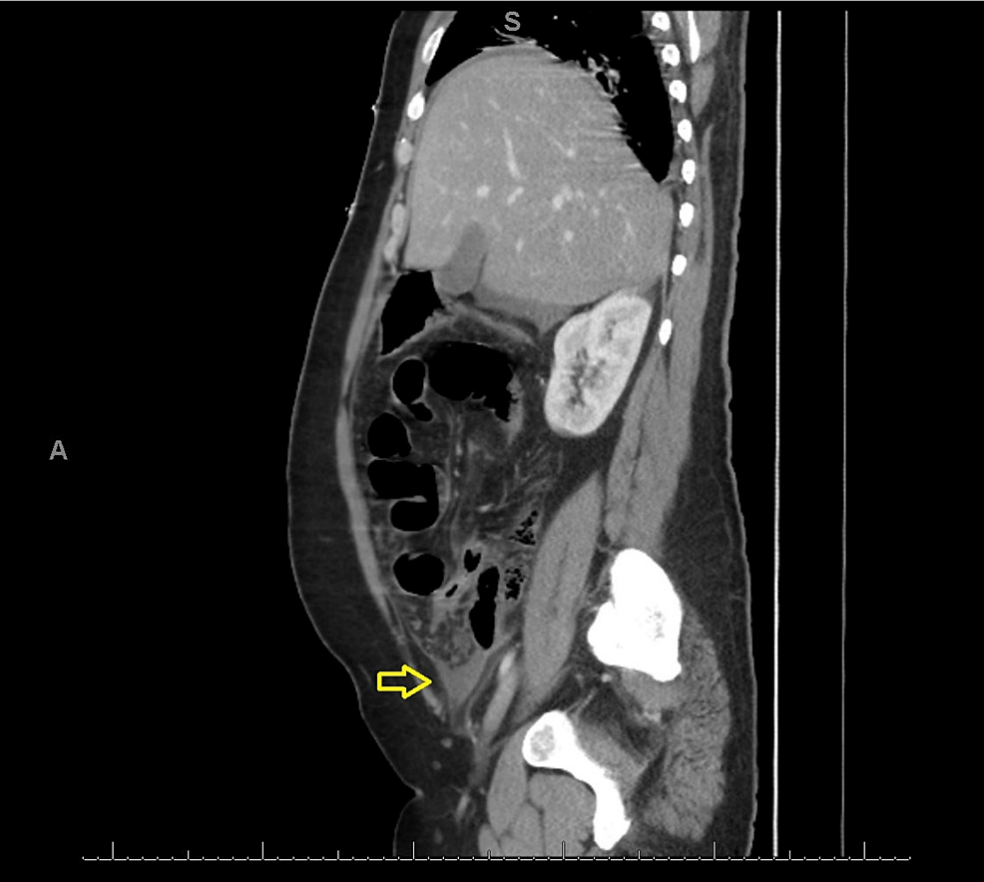
A sagittal view of an abdominal CT scan shows findings of perforated appendicitis. The area of pus collection is labeled with an arrow, and there is no extension of pus collection into the right inguinal canal.

Investigations showed leukocytosis (white blood cells (WBC): 12,000/µL). The CT scan of the abdomen and pelvis showed perforated appendicitis with an abscess and evidence of few free air pockets within free fluid in the abdomen and pelvis (Figures 1, 2).

The patient was immediately taken to the operating room for an exploratory laparotomy. A large amount of free purulent fluid was suctioned. The appendix was perforated at the tip and surrounded by a mass containing an abscess. The appendix was successfully removed, the abscess was drained, the abdomen was washed, and a drain was placed in the right lower abdomen. The patient was transferred to the surgical ward in stable condition.

**Figure 3:**
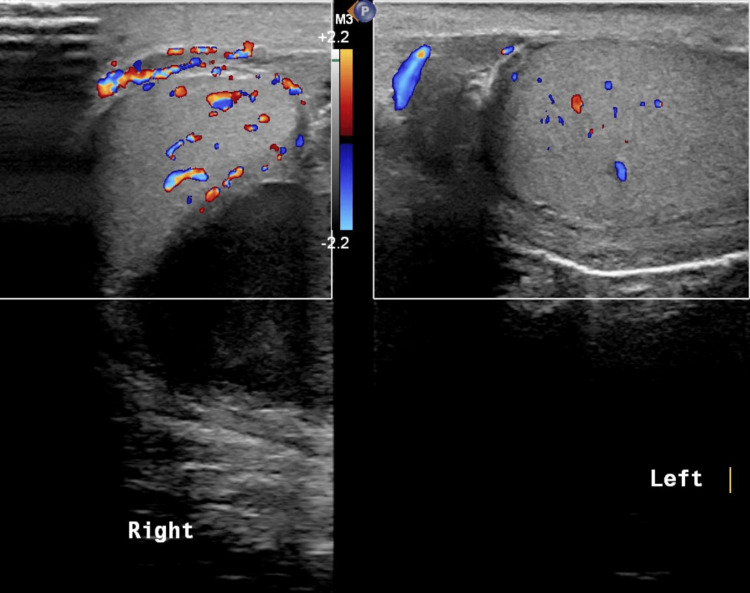
The scrotal ultrasound shows increased blood flow to the right testicle, with fluid collection around the right testicle.

On postoperative day two, he started to have right scrotal pain and swelling. On examination, he had mildly tender right hemiscrotal swelling with mild erythema. His WBC had significantly increased to 20,000/µL. The scrotal ultrasound (US) showed increased blood flow to the right testicle, with fluid collection around the right testicle (Figure 3).

The initial clinical impression was right epididymo-orchitis with reactive hydrocele. The patient was conservatively treated with IV antibiotics. His WBC continued to rise and reached 23,000/µL on postoperative day four. The pus culture, which was taken intraoperatively from the appendicular abscess, showed no growth. The patient tolerated a diet; his abdomen was soft and lax, the wound was clean, and the abdominal drain output was serous and minimal. The decision was taken to change the antibiotic to a wider-spectrum antibiotic.

**Figure 4:**
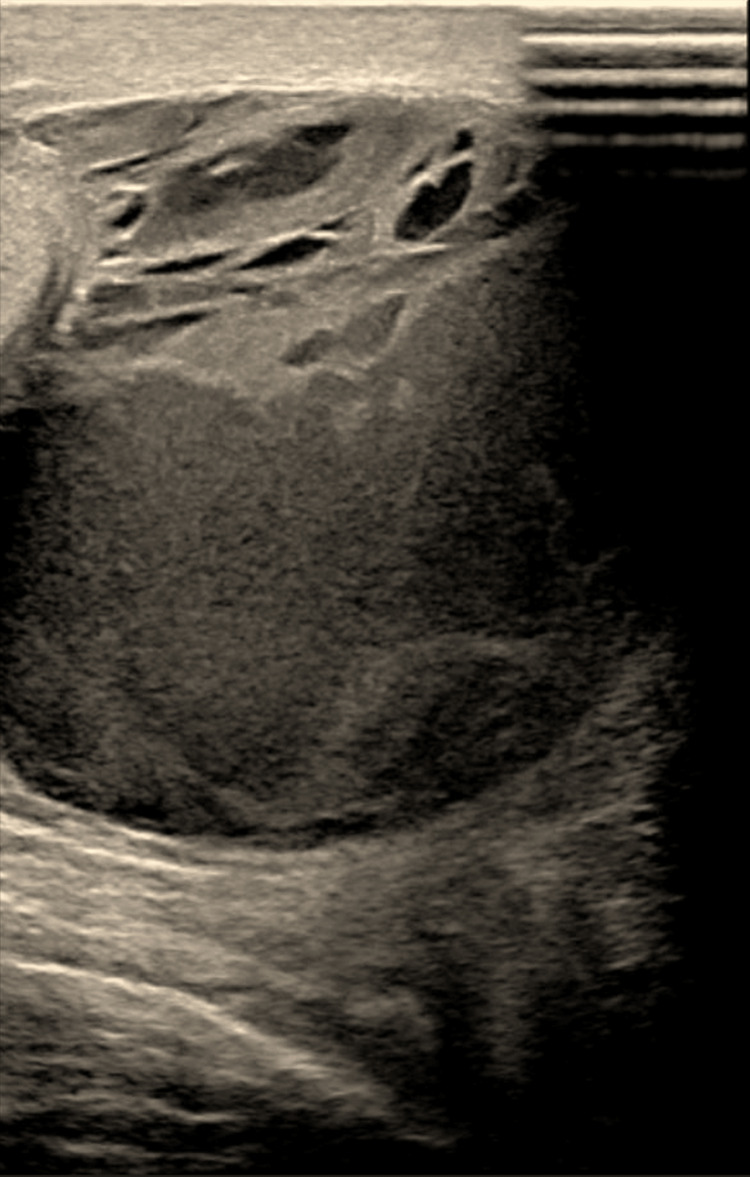
The scrotal ultrasound shows a loculated turbid fluid collection surrounding the right testicle with an internal thick hyperechoic septation; the findings are suggestive of a right-sided scrotal abscess.

His WBC improved and decreased to 11,700/µL on postoperative day seven, but he continued to complain of right scrotal swelling and pain. The scrotal US was repeated, and it showed loculated turbid fluid collection surrounding the right testicle with an internal thick hyperechoic septation. The findings were suggestive of a right-sided scrotal abscess (Figure 4).

The decision was taken to take the patient to the operating room for right scrotal exploration, and the patient agreed to surgery. Intraoperatively, it was found that pus had collected within the tunica vaginalis, which was multiloculated. About 50 ml of pus was drained, and debridement was done on the necrotic sloughs of the tunica vaginalis. The right testicle was viable, and no testicular abnormality was seen except for the attached superficial slough tissue, which was removed; plication was done on the remaining part of the tunica vaginalis; and a drain was placed in the right hemiscrotum. Pus was sent for culture and sensitivity.

Postoperatively, the patient did well; his WBC had been normalized to 8,100 /µL, drain output was nil, the drain was removed on postoperative day two, and he was discharged home in good condition. The pus culture, which was taken intraoperatively from the scrotal abscess, showed no growth.

## Discussion

A scrotal abscess following perforated appendicitis is a rare complication. It has been seen mostly in patients with patent processus vaginalis, and mainly seen in the pediatric age group. Two published articles have reviewed the previously reported cases in children and adolescents [3, 4]. There were a very limited number of cases reported among young adults, and our case report is the first one directed at the adult age group.

We performed a retrospective review of prior literature on scrotal abscess secondary to perforated appendicitis that discussed the clinical approach and treatment of scrotal pyocele post-appendectomy in all age groups, and then we selected the reports for patients aged ≥16 years. Children, by law, are those up to the age of 18. However, 16 years is the age at which a patient will be seen by a doctor for adults in the UK [5]. This is also what is usually accepted in many countries all over the world.

A retrospective literature review of published adult case reports showed seven cases in which the patient developed scrotal abscess after perforated appendicitis. The age of reported cases was 16, 17, 18, 19, 20, 25, and 30 years. The review is summarized in Table 1.

**Table 1:** Scrotal abscess in adult patients as a complication of perforated appendicitis RT: right; LT: left; appy: appendectomy; lap: laparoscopic; I&D: incision and drainage; PPV: patent processus vaginalis Postoperative days are shown in parentheses.

Author	Age (years)	Side affected	Surgery (primary)	Surgery (secondary)	Presence of PPV
Kollias et al. (1996) [6]	17	LT	Lap appy	Scrotal I&D (4)	-
Lantsberg et al. (1997) [7]	20	LT	Lap appy	Scrotal I&D (1)	-
Ng et al. (2002) [8]	25	RT	Open appy	Scrotal exploration, drainage of intraperitoneal abscess (5)	+
Lee et al. (2003) [9]	19	RT	Open appy	Scrotal exploration (3), drainage of retroperitoneal abscess	-
Shehzad et al. (2011) [10]	16	RT	Scrotal exploration and open appy	No further surgery	+
Shashidhara et al. (2014) [11]	30	RT	Right hemiscrotal exploration, exploratory laparotomy, and open appy	No further surgery	+
Wani et al. (2019) [12]	18	RT	Scrotal exploration, exploratory laparotomy, open happy, and drainage of intra-abdominal abscess	No further surgery	+

**Figure 5:**
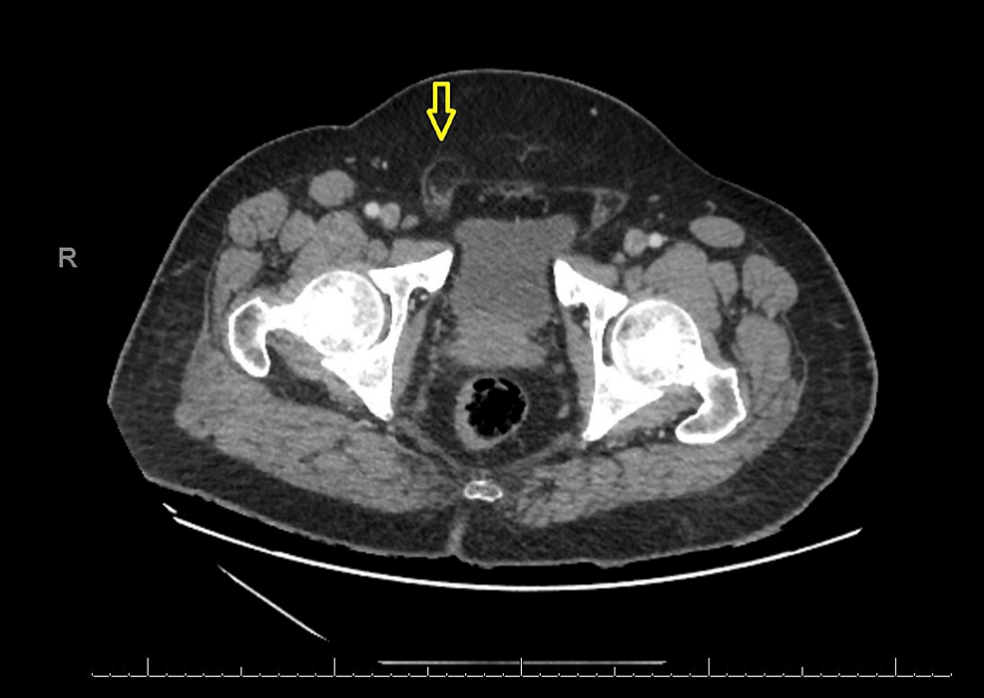
A CT scan shows prominent fat in the proximal part of the right inguinal canal, which suggests the presence of a partially patent processus vaginalis.

Scrotal pyocele secondary to perforated appendicitis can occur in the presence of a grossly patent processus vaginalis as well as in a grossly non-patent processus vaginalis. Passage of fluids through grossly non-patent processus vaginalis has been explained by many authors. This can be attributed to the microscopically permeable processus vaginalis in those with grossly non-patent processus vaginalis and some authors relate that to the presence of micro-perforations in the processus vaginalis that allow purulent material to pass into the inguinal region and scrotum [3]. Other authors have attributed that to the presence of an undiagnosed congenital inguinal hernia [7]. In our case, although we did not find patent processus vaginalis at the time of scrotal exploration, a preoperative CT scan showed prominent fat in the proximal part of the right inguinal canal, which suggests the presence of a partially patent processus vaginalis (Figure 5).

Cases that occur post-laparoscopic appendectomy have been attributed by some authors to the translocation of intra-abdominal bacteria through the closed processus vaginalis due to the higher insufflation pressure needed during laparoscopy [3]. Others have reported the occurrence of hydrocele post-laparoscopic cholecystectomy and have attributed the reopening of the obliterated processus vaginalis to high intra-abdominal pressure [13].

In our review, considering our case, 50% of patients were found to have an identifiable patent processus vaginalis intraoperatively, and the remaining 50% had no detectable patency of the processus vaginalis on gross inspection at the time of surgery. The right side was more affected (75%) than the left side (25%). Fifty percent of patients had no scrotal symptoms at the initial presentation, and they developed symptoms post-appendectomy and therefore needed another surgery. Fifty percent of patients had acute scrotum at the initial presentation, and they were found to have perforated appendicitis concomitant with scrotal pyocele.

## Conclusions

This review emphasizes the importance of considering scrotal pyocele in any patient with acute scrotum post-appendectomy, regardless of the patient's age, the affected side, or the presence or absence of identifiable patent processus vaginalis. Early diagnosis is important to avoid complications. Ultrasound and CT scans can help in the diagnosis, as they may demonstrate the presence of a scrotal abscess communicating with the abdomen through a patent processus vaginalis. Treatment entails urgent drainage of both scrotal and abdominal abscesses, together with a course of antibiotics.
